# Gesture during math instruction specifically benefits learners with high visuospatial working memory capacity

**DOI:** 10.1186/s41235-020-00215-8

**Published:** 2020-06-09

**Authors:** Mary Aldugom, Kimberly Fenn, Susan Wagner Cook

**Affiliations:** 1 Department of Psychological and Brain Sciences, University of Iowa, Iowa City, IA 52242 USA; 2 Department of Psychology, Michigan State University, East Lansing, MI USA

**Keywords:** Gesture, Math learning, Working memory

## Abstract

**Background:**

Characteristics of both teachers and learners influence mathematical learning. For example, when teachers use hand gestures to support instruction, students learn more than others who learn the same concept with only speech, and students with higher working memory capacity (WMC) learn more rapidly than those with lower WMC. One hypothesis for the effect of gesture on math learning is that gestures provide a signal to learners that can reduce demand on working memory resources during learning. However, it is not known what sort of working memory resources support learning with gesture. Gestures are motoric; they co-occur with verbal language and they are perceived visually.

**Methods:**

In two studies, we investigated the relationship between mathematical learning with or without gesture and individual variation in verbal, visuospatial, and kinesthetic WMC. Students observed a videotaped lesson in a novel mathematical system that either included instruction with both speech and gesture (Study 1) or instruction with only speech (Study 2). After instruction, students solved novel problems in the instructed system and transfer problems in a related system. Finally, students completed verbal, visuospatial, and kinesthetic working memory assessments.

**Results:**

There was a positive relationship between visuospatial WMC and math learning when gesture was present, but no relationship between visuospatial WMC and math learning when gesture was absent. Rather, when gesture was absent, there was a relationship between verbal WMC and math learning.

**Conclusion:**

Providing gesture during instruction appears to change the cognitive resources recruited when learning a novel math task.

## Significance statement

This work is a collaborative effort to better understand the individual difference factors that predict mathematical learning with gesture. One of the laboratories has expertise in gesture and mathematical learning and the other studies individual differences in learning. This work was supported by a collaborative grant from the National Science Foundation, which is interested in uncovering the factors that contribute to successful STEM learning and education. It is well-established that when learners observe gesture, learning is enhanced (Cook, Duffy, & Fenn, 2013; Cook, Friedman, Duggan, Cui, & Popescu, 2016); however, the mechanisms underlying this effect remain largely unexplored. We used our combined expertise to better understand how variation in working memory capacities across learners might support mathematical learning, with a focus on visuospatial working memory capacity (WMC). Understanding how gesture works at the individual level can improve our theories of gesture processing and is also important for capitalizing on gesture to enhance mathematical education. Our findings reveal a link between visuospatial working memory and mathematical learning with gesture at instruction, and knowledge of this link could be used to enrich mathematical learning in the real world.

## Background

Many children fail to achieve proficiency in math. In the United States, only 40% of students in grade four, 34% of students in grade eight, and 25% of students in grade 12 were above proficient in mathematics at both public and private schools in 2017 (National Assessment of Educational Progress: National Center for Education Statistics (NCES), 2017). Several factors likely contribute to students’ difficulties in acquiring expected levels of math proficiency; here we focus on two factors that have previously been shown to influence math learning: instruction that includes hand gesture and visuospatial, verbal, and kinesthetic WMC in the learners.

### Working memory and math learning

Working memory processes are known to support math learning (e.g. Alloway & Alloway, 2010). Baddeley developed a modality-specific model of working memory, with two modality specific subsystems, a *phonological loop*, which stores linguistic information, and a *visuospatial sketchpad*, which stores visual and spatial information (Baddeley & Hitch, 1974; Baddeley, 2003; although see Engle, 2002 and Cowan, 1999 for alternative accounts of working memory).

Modality-specific working memory capacities are related to math learning. The capacity of verbal working memory predicts various types of mathematical success, across time and across development (Alloway & Alloway, 2010; Gathercole, Pickering, Knight, & Stegmann, 2004; Noël, Seron, & Trovarelli, 2004; Passolunghi, Vercelloni, & Schadee, 2007). For example, children’s verbal working memory skills at age five are correlated with their academic achievement in mathematics 6 years later (Alloway & Alloway, 2010). Visuospatial working memory also predicts mathematical success (Kyttälä, 2008; van der Ven, van der Maas, Straatemeier, & Jansen, 2013). Further, some studies have measured both verbal and visuospatial WMC and find that both predict success in mathematics (Jarvis & Gathercole, 2003; Swanson & Beebe-Frankenberger, 2004), although this pattern is not always seen (St. Clair-Thompson & Gathercole, 2006).

Spatial ability is also a significant predictor of mathematical ability in both children and adults (Cheng & Mix, 2014; Lubinski, 2010; Wai, Lubinski, & Benbow, 2009). Measures of spatial ability most often encompass spatial visualization and spatial reasoning, and it is possible that visuospatial working memory and spatial abilities overlap as cognitive constructs (Miyake, Friedman, Rettinger, Shah, & Hegarty, 2001).

### Gesture and math learning

Although WMC is considered to be a fixed capacity, characteristics of instruction can influence how working memory resources are used during learning (Paas, Renkl, & Sweller, 2004). According to cognitive load theory, instruction that increases the capacity of learners to hold relevant information in working memory should increase learning (Sweller, 2010). One hypothesis is that multimodal instruction facilitates learning by allowing learners to efficiently use available working memory resources (Mayer, 2005; Mayer & Moreno, 2003; Mousavi, Low, & Sweller, 1995).

Indeed, multimodal instruction that includes visual hand gestures along with auditory speech has been shown to improve mathematical learning. Gestures are hand movements that spontaneously accompany speech, that are related to speech both semantically and temporally, and that do not serve any other known function. There are several different types of hand gestures that emerge across development and that are tightly coupled with language (see Capone & McGregor, 2004 for a review). Here we focus mostly on deictic gestures, which index specific objects or items in the environment and are often used to direct attention to the item being referenced (Bangerter & Louwerse, 2005). For example, in the current study, a math instructor simultaneously points at and audibly names each element of a mathematical equation as she explains a procedure for solving the problem. These sorts of gestures are frequent in math instruction (Alibali et al., 2014; Flevares & Perry, 2001).

Observing gesture during instruction enhances math learning, for adults as well as for children (e.g. Cook et al., 2013; Cook et al., 2016; Hendrix, Fenn, & Cook, 2018; Ping & Goldin-Meadow, 2008; Valenzeno, Alibali, & Klatzky, 2003). Furthermore, the beneficial effect of the observation of gesture on performance is pronounced for more challenging problem-solving tasks (Hou & So, 2017).

The mechanism by which gesture increases learning is not known. From the perspective of cognitive load theory, there are two possibilities for how multimodal instruction might reduce demand on working memory. One possibility is that multimodal instruction, because it involves separate systems for each modality, allows learners to capitalize on working memory resources that would otherwise not be recruited. On this account, gesture might provide a mechanism for involving visuospatial and kinesthetic working memory in learning (Wu & Coulson, 2007, 2011) that would otherwise be subserved only by verbal working memory. An alternative is that multimodal instruction might improve the efficiency with which information is encoded without necessarily recruiting additional resources. Gestures allow instructors to direct attention to the features of the problem. By providing a tool for signaling relevant information during instruction (Mayer, 2002), gestures may enhance encoding of relevant information into working memory.

These two accounts make different predictions about which learners should benefit most from instruction that includes gesture. If gestures recruit additional resources, perhaps visuospatial resources, then gesture should support learners with greater visuospatial WMC by allowing these learners to capitalize on their greater resources. Alternatively, if gestures facilitate encoding, gesture should enhance learning for learners with lower relevant WMC, by allowing these learners to make better use of their limited resources.

Understanding the working memory resources that support learning from gesture has implications for identifying the mechanisms underlying gesture processing more generally. Although gestures co-occur with language, they represent information visually and spatially and so they may engage spatial processing during linguistic communication. Indeed, some theories of gesture suggest that they are particularly important during spatial communication (Hostetter & Alibali, 2007) while others emphasize the relationship between gesture and language (McNeill, 1992; Rowe & Goldin-Meadow, 2009). If gestures are coded spatially, we would expect gesture processing to be related to spatial WMC (. Alternatively, if gestures function to influence the processing of concurrent linguistic information, we would expect gesture processing to be related to verbal WMC.

The available evidence suggests that sensitivity to gestures may depend on available visuospatial and/or kinesthetic working memory resources. For example, in a priming task that included both speech and gesture, individuals with higher spatial WMC showed more sensitivity to information in gesture, while those with higher verbal WMC showed more sensitivity to information in speech (Özer & Göksun, 2019). Similarly, Wu and Coulson (2014b) examined the role of verbal and visuospatial WMC in gesture comprehension and found that individuals with high visuospatial WMC were more sensitive to gesture than those with low visuospatial WMC. In a separate study, these researchers examined the relationship between performance on a gesture comprehension task and kinesthetic working memory, a novel subsystem of working memory which is postulated to be responsible for the storage and manipulation of bodily movements (Wu & Coulson, 2015). Individuals with high kinesthetic WMC were more sensitive to gestures and were better able to inhibit information from irrelevant gestures (Wu & Coulson, 2015). Together, these findings suggest that, in perception, sensitivity to gesture may depend on available visuospatial and/or kinesthetic resources, benefitting those with high capacity in relevant modalities.

However, the resources involved in learning from gesture may be distinct from those involved in understanding through gesture. In this article, we examine the relationships between math learning with gesture present or absent at instruction, visuospatial WMC, verbal WMC, and kinesthetic WMC. In Study 1, we examined performance on an abstract mathematical task with gesture present at instruction. We used only a single instructional condition in order to increase power to detect relationships between WMC and learning. After finding such relationships in Study 1, in Study 2, we examined performance on an abstract mathematical task with gesture absent at instruction using a new sample of participants. We then combined findings from Study 1 and Study 2 to compare patterns across instructional conditions.

### Predictions

The findings from Wu and Coulson (2014b, 2015) and from Özer and Göksun (2019) reveal that gestures load on visuospatial and kinesthetic WMC. However, these studies investigated action words and discourse processing, not learning. If these findings generalize to instructional contexts, we would expect the availability of gesture during instruction might allow learners to use visuospatial and kinesthetic WMC that would otherwise not be engaged during instruction. If so, then adding gesture to instruction should improve learning for individuals with high visuospatial and kinesthetic WMC.

To test these predictions, Study 1 examined the relationship between individual differences in verbal, visuospatial, and kinesthetic WMC and learning when instruction includes gesture. Participants watched video instruction on a new mathematical system where the instructor used both speech and gesture. Following instruction, participants completed a posttest and a transfer test to assess learning. They then completed a visuospatial working memory task, a verbal working memory task, and a kinesthetic working memory task. Finally, participants completed an abbreviated mathematical anxiety rating scale and gesture attitudes questionnaire. Our goal in Study 1 was to assess how learning a novel mathematical concept with both speech and gesture at instruction relate to visuospatial, verbal, and kinesthetic WMC.

### Study 1

The objective of Study 1 was to examine the relationship between individual differences in visuospatial, verbal, and kinesthetic WMCs and learning mathematical equivalence with gesture. Approval was obtained from the Institutional Review Board prior to data collection.

### Participants

Seventy-five University of Iowa undergraduates participated in the study. Eleven participants were excluded from the final analyses. Participants were excluded for being non-native English speakers (*n* = 2), technical errors (*n* = 3), for not performing above chance in the learning task (n = 2), or because they did not have available ACT scores (*n* = 4). Thus, only 64 native English speakers were included in the analyses (35 male, 29 female). We determined this sample size a priori, based on simulations of our design and assumptions about effect sizes derived from prior research (Marstaller & Burianová, 2013; Wu & Coulson, 2015). We also preregistered our analytic approach with the Open Science Framework based on our expectations. Participants received course credit for participation.

### Materials

#### Abstract Mathematical Equivalence Task

**Fig. 1:**
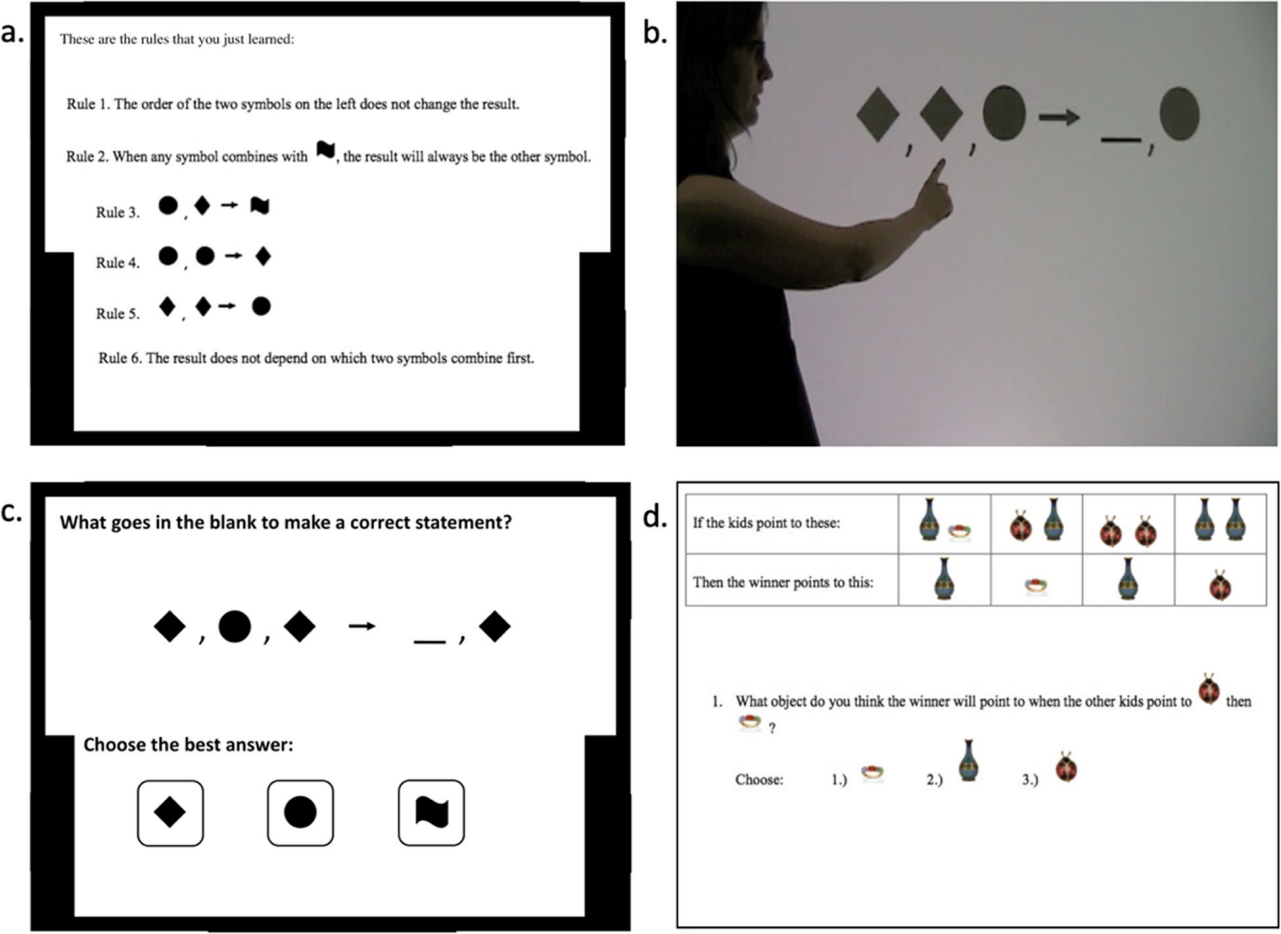
Example images from the abstract mathematical task. **a** The summary depiction of the six rules presented to participants prior to instruction. **b** Screenshot from an example instructional video with gesture. **c** An example problem from the posttest, where the correct answer is squiggle. **d** An example problem from the transfer task

To assess learning with gesture, we used an abstract Mathematical Equivalence Task modified from Hendrix et al., 2018 (originally adapted from Kaminski, Sloutsky, & Heckler, 2008). This is a completely novel mathematical task, created for studying math learning in the laboratory. The task follows a system of modular arithmetic and requires students to learn to solve problems in a commutative group of order three, a mathematical system operating over shapes (diamond, circle, and squiggle) (see Fig. 1). We did not include a pretest because participants had no prior experience with the stimuli and the rules for combining them in our abstract math system. As such, they had no knowledge of the meaning of the symbols or the ways in which to combine them.

Participants first learned six rules for combining the three shapes in this novel mathematical system. Rules were presented one at a time in written format on a computer screen. All six rules are displayed in Fig. 1a. Participants had an unlimited amount of time to read each rule and chose when to proceed. After each rule was presented, participants answered one or two practice problems before seeing the next rule. These practice problems all tested simple equations that required participants to combine two shapes and calculate the result. These problems tested participants’ understanding of the preceding rule. Participants received feedback if they selected an incorrect response and were required to repeat the question until they selected the correct response. After viewing the rules and solving the practice problems, participants were shown all six rules together (Fig. 1a) and had as much time as needed to read and reflect on the six rules before proceeding to the instructional videos.

Participants then viewed six video-recorded explanations that described how to solve more complex problems in the symbol system. The problems used during instruction were based on math problems used to study the concept of equivalence in younger children and required participants to apply the rules to problems with five symbols; there were three symbols on the left side of the problem and one symbol and one blank space (a missing symbol) on the right sides of the problem. The instructor explained how to solve the problem to find the symbol that belonged in the blank space. The videos included both speech and gesture and ranged from 13 to 33 seconds. All gestures used in the instructional videos were deictic gestures; the instructor in the video pointed to the shapes in the equation as she elaborated mathematical equivalence problem-solving strategies (see Fig. 1b). In each video, the instructor points to each shape and explains how the shapes combine with each other as well as what shape each side of the question reduces to. She then gives the answer for which shape belongs in the blank space (access https://osf.io/wh92e/ for instructional videos).

After each instructional video, participants solved a practice problem, similar in form to the problem presented in the video. Participants could not move forward until they selected the correct response. Following instruction, participants were given a posttest (27 questions) and a transfer test (12 questions) to assess learning and generalization. All of the problems on the posttest and transfer test were novel; these problems did not appear during training. All posttest and transfer test problems were scored as correct or incorrect, or 1 or 0, respectively. The transfer task followed a similar mathematical structure as the abstract math task, but it used different objects, requiring participants to generalize their knowledge beyond the learning context (see Fig. 1d). Unlike in the posttest, however, during the transfer test, the rules for combining symbols in this new system were visible during problem solving on each trial, so participants did not need to keep this information in memory. The top portion of the image in Fig. 1d appeared on each transfer test problem. Cronbach’s alpha for the posttest was 0.86 and for the transfer test was 0.74. Thus, both tests demonstrated sufficient internal consistency to serve as individual measures of learning.

#### Visual Patterns Task

To assess visuospatial WMC, we used an adaptation of the Visual Patterns Task (Chu & Kita, 2011; adapted from Della Sala, Gray, Baddeley, & Wilson, 1997). Test-retest reliability of the Visual Patterns Task is 0.75 (Della Sala et al., 1997).

Participants viewed patterns of white and black blocks, presented for 3 seconds each. Immediately after the presentation of the pattern, the patterns of blocks were replaced with letters in every block, and participants were prompted to verbally recall the letters corresponding to the black blocks that were previously shown. Participants did not need to remember these letters, as the letters were visible throughout recall, serving to provide an efficient way of referencing the spatial locations in the grid. Spans ranged from seven to eleven black blocks with five trials at each level and an equal number of white and black blocks at each level. Responses were video recorded and scored online and offline.

#### Sentence Span Task

To assess verbal WMC, we used an adaptation of the Reading Span Task (Waters, Caplan, & Hildebrandt, 1987). The Reading Span Task is considered the standard task for assessing verbal WMC. Test-retest reliability composite Z-score measures were calculated separately for cleft subject sentence (*r*_z_ = 0.75) and for subject-object sentences (*r*_z_ = 0.83), demonstrating high test-retest reliability (Waters & Caplan, 1996).

Participants viewed a series of sentences and made judgments about each sentence. At the end of each trial (two to eight sentences), participants were prompted to verbally recall the last word of each sentence in the order that they had been shown. Spans ranged from two to eight sentences with five trials at each level. Responses were video recorded and scored online and offline.

#### Movement Span Task

To assess kinesthetic working memory, we used an adaptation of the Movement Span Task that has previously been related to sensitivity to information from gesture (Wu & Coulson, 2014a; Wu & Coulson, 2015). Analyses demonstrate a high reliability estimate of α  = 0 .95 (Wu & Coulson, 2014a).

Participants viewed a series of hand and arm movements presented on video. At the end of each trial, participants were prompted to replicate the movements with as much detail as possible. Spans ranged from one to five movements with three trials at each level. Responses were video recorded, and movements were coded and scored offline. Single points were given for each movement that was replicated correctly, half points were given for each movement that reflected the target movement, but had slight deviations, and no points were given to movements that did not reflect any movements within a span. Points did not depend on the order in which participants recalled each movement within a span. Because we implemented a specific coding system for the movements, we assessed reliability; the intercoder agreement for scoring kinesthetic working memory span was 99%.

#### Composite ACT Score

Composite ACT score was obtained from university records and was used to control for cognitive ability. Performance on the ACT correlates highly with independent measures of general intelligence (Koenig, Frey, & Detterman, 2008). The median reliability estimate for the composite ACT score is 0.97. We expected that composite ACT score would positively predict learning in our novel math learning task.

#### Abbreviated Math Anxiety Rating Scale (A-MARS)

The A-MARS was included at the end of the study as an exploratory measure of mathematical anxiety (Alexander & Martray, 1989). The A-MARS is a 25-item questionnaire with a 5-point Likert scale ranging from “Not At All” to “Very Much”; participants were asked to indicate their level of anxiety in mathematical-relevant scenarios. Analyses demonstrate a high reliability coefficient of α  = 0 .98.

#### Gesture Attitudes Questionnaire

A Gesture Attitudes Questionnaire was included at the end of the study as an exploratory measure (Nathan, Yeo, Boncoddo, Hostetter, & Alibali, in press). The Gesture Attitudes Questionnaire is a 16-item questionnaire with a 5-point Likert scale ranging from “Strongly Disagree” to “Strongly Agree”; participants were asked to indicate their level of agreement to specific statements about the function of gesture during communication.

### Procedure

Participants were run individually, and the experimenter was in the testing room throughout the session. The study was conducted in a fixed order; all participants completed each task in the order in which they were described previously and completed a short participant information questionnaire at the end of the study.

## Results

We assessed learning by measuring problem-solving accuracy on the posttest and transfer test. We then modeled the extent to which visuospatial, verbal, and kinesthetic working memory predicted learning. Composite ACT score was included as a covariate. All variables were normally distributed with the exception of kinesthetic working memory, which was positively skewed. Working memory spans, and composite ACT scores were all standardized prior to analysis.

To assess multicollinearity, we first examined the bivariate correlations among predictors. The correlations among the three working memory measures were relatively weak (*r* = .27 for visuospatial and verbal working memory, *r* = .30 for kinesthetic and visuospatial working memory, and *r* = .26 for verbal and kinesthetic working memory), suggesting that the various forms of WMC were unconfounded in our sample. To assess the potential impact of multicollinearity on our statistical models, we calculated variance inflation factor (VIF) values for all predictors. All values were under 2.50 (visuospatial working memory = 1.62, verbal working memory = 1.74, kinesthetic working memory = 1.27, and composite ACT score = 1.57), suggesting that the level of multicollinearity in our data was low. The correlations between composite ACT score and verbal working memory (*r* = .36) and ACT and kinesthetic working memory (*r* = .16) were also weak. There was a fairly large correlation (*r* = .61) between ACT and visuospatial working memory. ACT was included as a control variable and was entered into the models before our predictor variables. Thus, any effects of our working memory measures were interpreted as above and beyond effects of composite ACT score.

The mean performance on the posttest was 0.75 (range: 0.30–1, *SD* = 0.21) and mean performance on the transfer test was 0.61 (range: 0.17–1, *SD* = 0.24), indicating that participants were successful in learning from our instruction. We used a generalized logistic mixed-effect model to account for variability across subjects and difficulty of problems. We modeled the log odds of correctly solving each problem from composite ACT score, verbal working memory span, visuospatial working memory span, and kinesthetic working memory span. We also included participant and problem intercepts as random effects. Our preregistered model (available in the Supplemental Material) included all higher-order interactions, however there were no significant interactions and there was no evidence to suggest that removing the higher-order interactions significantly decreased model fit (Post: χ^2^(4) = 1.19, *p* = .88; Transfer χ^2^(4) = 5.90, *p* = .21), and so we report the simpler model without any interactions here.

**Fig. 2:**
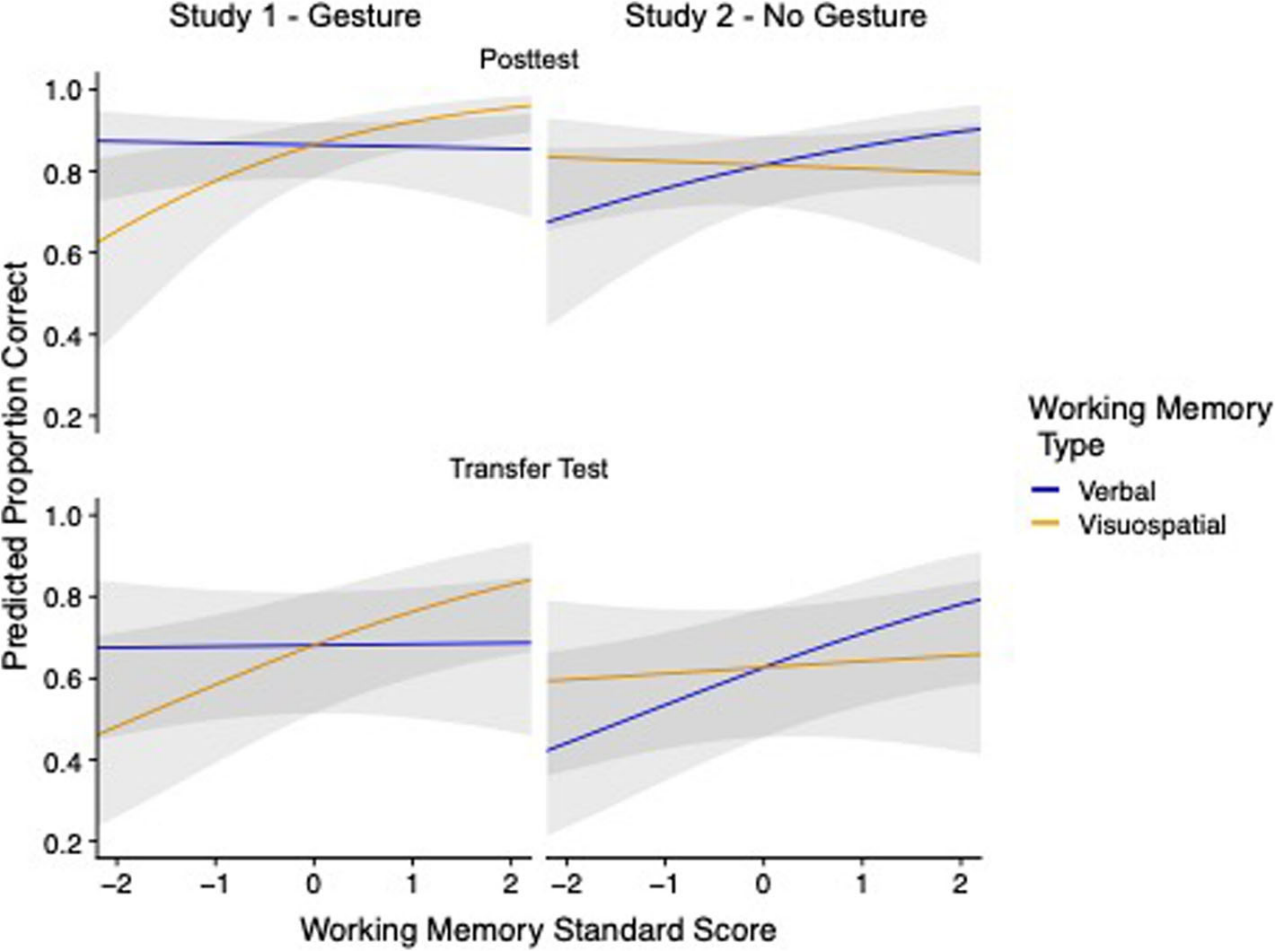
Model predictions from the combined analysis of Study 1 (*left column*) and Study 2 (*right column*) for the posttest (*top row*) and the transfer test (*bottom row*). The multilevel logistic models predicted posttest or transfer test performance from two-way interactions between gesture group and verbal working memory span, and gesture group and visuospatial working memory span, with composite ACT score as a covariate, and random intercepts for participant and problem. For graphical purposes, performance was separately predicted from verbal and from visuospatial working memory capacities while holding all other variables in the model constant. Error bars represent the 95% confidence interval of the predictions

The findings for posttest and transfer test were highly similar to one another. For posttest performance, composite ACT was a significant predictor (β = 0.86, *z* = 3.62, *p* < 0.001) and visuospatial working memory was a marginal predictor (β = 0.42, *z* = 1.79, *p* = 0.074; see Fig. 2). Kinesthetic and verbal working memory were not significantly associated with performance (kinesthetic: β = 0.075, *z* = 0.36, *p* = 0.72; verbal: β = − 0.11, *z* = − 0.57, *p* = 0.57). For transfer test performance, both composite ACT (β = 0.47, *z* = 2.39, *p* = 0.017) and visuospatial working memory (β = 0.43, *z* = 2.17, *p* = 0.030) were significant predictors. Kinesthetic and verbal working memory were not significantly associated with performance (kinesthetic: β = 0.082, *z* = 0.48, *p* = 0.63; verbal: β = 0.011, *z* = 0.067, *p* = 0.95).

We also explored the relationship between scores on the A-MARS and performance on the abstract math task and the three working memory measures. These analyses, reported in the Supplementary Materials, revealed that math anxiety did not account for the relationship between visuospatial WMC and learning with gesture. Finally, we explored whether gesture attitudes were related to learning with gesture using the Gesture Attitudes Questionnaire; however, there was no evidence that the two were related (see Supplementary Materials).

Experiment 1 provided evidence that learning mathematical equivalence with gesture is significantly related to visuospatial WMC, particularly for transfer performance. However, it is possible that this pattern simply reflects demands of the abstract math task, which requires operating over visual shapes, rather than the presence of gesture at instruction. Because all participants viewed instruction with gesture, Study 1 cannot discern these two possibilities. Accordingly, we conducted a follow-up study, where participants learned the same material without accompanying gesture.

### Study 2

Study 2 was identical to Study 1 except that, in the video instruction, the instructor did *not* gesture during instruction (the instructors hands stayed at her side; Fig. 1b). The participants in Study 2 were different than those in Study 1; however, all participants from Study 1 and Study 2 were University of Iowa undergraduates concurrently enrolled in Elementary Psychology. Approval was obtained from the Institutional Review Board prior to data collection.

### Participants

Sixty-eight University of Iowa undergraduates participated in the study. Of these, four participants were excluded from the final analyses, either for not performing above chance in the abstract mathematical learning task (*n* = 1) or because composite ACT scores were not available (*n* = 3). Thus, 64 native English speakers were included in the analyses (17 male, 47 female). As in Study 1, we determined this sample size a priori, based on simulations of our design and assumptions about effect sizes derived from prior research (Marstaller & Burianová, 2013; Wu & Coulson, 2015), and we preregistered our analytic approach with the Open Science Framework based on our expectations. Participants received course credit for participation.

## Results

Our analytic approach was identical to Study 1. The bivariate correlations among the three working memory measures were again weak (*r* = .31 for visuospatial and verbal working memory, *r* = .29 for kinesthetic and visuospatial working memory, and *r* = .13 for verbal and kinesthetic working memory). Again, we calculated VIF values for all predictors to ensure no issues regarding multicollinearity, and all values were under 2.50 (visuospatial working memory = 1.59, verbal working memory = 1.65, kinesthetic working memory = 1.50, and composite ACT score = 1.71). Similar to Experiment 1, there were small correlations between ACT and verbal working memory (*r* = .39) and ACT and kinesthetic working memory (*r* = −.04), and a moderate correlation (*r* = .51) between ACT and visuospatial working memory. All variables were normally distributed with the exception of kinesthetic working memory, which was positively skewed. The three working memory measures and composite ACT scores were all standardized prior to analysis.

Mean performance on the posttest was 0.74 (range: 0.37–1, *SD* = 0.17), and on the transfer test was 0.59 (range: 0.17–1, *SD* = 0.22). We again focused on exploratory models with no interaction terms as there was no evidence to suggest that the higher-order interactions included in our preregistered analysis improved model fit (Post: χ^2^(4) = 2.16, *p* = .71; Tran χ^2^(4) = 6.38, *p* = 0.17; full model available in Supplementary Materials).

Again, the pattern of results was similar across the posttest and the transfer test. For posttest performance, composite ACT was a marginal predictor (β = 0.40, *z* = 1.84, *p* = 0.066) and verbal working memory was a significant predictor (β = 0.42, *z* = 2.03, *p* = 0.042; see Fig. 2). The effects of visuospatial working memory (β = 0.043, *z* = − 0.20, *p* = 0.84) and kinesthetic working memory (β = 0.23, *z* = 1.32, *p* = 0.19) were not significant. For transfer test performance, both ACT (β = 0.66, *z* = 3.73, *p* < 0.001) and verbal working memory (β = 0.36, *z* = 2.27, *p* = 0.023) were significant predictors. The effects of visuospatial working memory (β = 0.017, *z* = 0.099, *p* = 0.92) and kinesthetic working memory (β = 0.138, *z* = 0.19, *p* = 0.84) were not significant.

As in Study 1, we explored the relationship between math anxiety and gesture attitudes and performance on the abstract math test and on performance on our three working memory measures. Again, the analyses revealed that neither math anxiety nor gesture attitudes accounted for the relationship between verbal WMC and learning without gesture (see Supplemental Materials).

This pattern of results suggests that composite ACT score and *verbal* working memory significantly predict mathematical learning after instruction without gesture. Thus, the group of participants that were instructed without gesture did not show the same pattern observed in Experiment 1; when instruction included gesture, ACT and *visuospatial* working memory were associated with performance.

To further examine the relationship between learning in each instructional condition and WMC, we combined the data from both studies and conducted exploratory analyses including instructional group (gesture or no gesture) as an additional predictor. Because none of our previous analyses revealed a relationship between kinesthetic working memory and learning on either test, we did not include kinesthetic working memory in the combined analysis. Additionally, because model comparison did not support inclusion of interactions between forms of WMC, we report only the findings from the models predicting posttest and transfer performance from interactions between instructional group and visual and instructional group and verbal WMC.

Because the two samples were collected at separate times, we compared them. There were no differences in overall performance by group on any of our main tasks or measures (see Table 1). However, there was a significant difference in the gender distribution of the two samples (χ^2^(1) = 10.49, *p* < .01). We therefore examined the relationship between gender and learning; however, model comparison did not suggest that gender was related to learning or that the difference in gesture composition could account for differences in the patters across the two studies (see Supplemental Materials).

**Table 1:** Mean performance on measures by group

Group	Posttest performance	Transfer test performance	Composite ACT score	VSWM	VWM	KWM	Math anxiety
Gesture	0.75	0.61	26.1	14.0	4.2	2.5	51.5
No gesture	0.74	0.59	27.0	13.3	4.3	2.6	56.7

We modeled the log odds of correctly solving problems from two-way interactions between gesture group and verbal working memory span, and gesture group and visuospatial working memory span, with composite ACT score as a covariate. The gesture group served as the reference group in our coding scheme.

Again, the patterns across posttest and transfer were highly similar. In the combined posttest analysis, there was a positive main effect of composite ACT score on accuracy (β = 0.58, *z* = 3.67, *p* < .001). There was not a significant effect of instructional group (β = − 0.37, *z* = − 1.45, *p* = 0.15). There was also a positive main effect of visuospatial working memory on posttest accuracy (β = 0.61, *z* = 2.93, *p* < 0.01). This pattern was qualified by a significant negative interaction between gesture group and visuospatial WMC (β = − 0.67, *z* = − 2.52, *p* = 0.012), revealing that, in the no-gesture group, the association between visuospatial working memory and performance was significantly attenuated. This finding demonstrates that visuospatial working memory is significantly related to learning when gesture is present, but not when gesture is absent. The main effect of verbal working memory (β = − 0.038, z = − 0.21, *p* = 0.83) and the interaction of verbal working memory with group (β = 0.38, *z* = 1.42, *p* = 0.16) were not significant.

We then analyzed transfer test performance using the same model structure. There was again a positive main effect of composite ACT score (β = 0.55, *z* = 4.26, *p* < .001) and no main effect of instructional group (β = − 0.24, *z* = 1.16, *p* = 0.25). There was a positive main effect of visuospatial working memory (β = 0.41, *z* = 2.48, *p* = 0.013), and a trend for a negative interaction between group and visuospatial working memory, although the coefficient was not significant (β = − 0.35, z = − 1.64, *p* = 0.10). There was not a main effect of verbal working memory (β = 0.013, *z* = 0.089, *p* = 0.93), but there was a marginal interaction between gesture group and verbal working memory (β = 0.36, *z* = 1.71, *p* = 0.088). Thus, for transfer test performance, the pattern suggested a trend that visuospatial WMC was related to performance in the gesture group but not in the no-gesture group. Furthermore, verbal WMC was related to performance in the no-gesture group, but not related to performance in the gesture group.

We considered potential confounders. We examined if participants with a stronger mathematical background were responsible for the differential effects. Our effects did not differ after removing participants who self-reported majoring in math, engineering, marketing, or finance (*n* = 31), demonstrating that these effects were not driven by participants with greater mathematical knowledge (see Supplemental Materials). We also considered if differences in study time could explain our findings; however, including study time as a covariate in our analyses did not change the pattern of performance (see Supplemental Materials).

## Discussion

This study reveals that the working memory resources associated with learning in a novel math task vary depending on the nature of the instruction that is provided. In Study 1, when gesture was present at instruction, individuals with higher *visuospatial* WMC performed better on the abstract math task compared with individuals with lower visuospatial WMC, even after controlling for composite ACT score. In Study 2, when gesture was not present at instruction, there was no evidence that posttest performance was related to visuospatial WMC. Instead, individuals with higher *verbal* WMC performed better, even after controlling for ACT. Findings from our exploratory analyses combining the data from the two studies provide additional evidence that the pattern of association varied across the two instructional groups. Importantly, because the same abstract mathematical task was used in both the gesture and no-gesture conditions, these findings cannot be due to specific demands of the mathematical task but rather must reflect characteristics of the instruction, which varied across groups.

Individuals with high visuospatial working memory did not benefit from this capacity when instruction did not include gesture, but they showed enhanced learning when instruction did include gestures, suggesting that gestures may be encoded and processed in visuospatial working memory. Although gestures co-occur with speech, they present information visuospatially, and so they must enter the processing system visuospatially. The finding that learning with gesture is related to visuospatial WMC suggests that visuospatial characteristics of gesture may continue to be important as language processing unfolds (Wu & Coulson, 2007, 2011). These findings suggest that gestures can function to engage cognitive resources that would otherwise not support learning.

Our initial hypothesis was that kinesthetic working memory would predict learning with gesture at instruction, given prior work finding that individuals with high kinesthetic WMC are more sensitive to gesture (Wu & Coulson, 2015). However, there was no evidence for a relation between kinesthetic WMC and learning in either instructional condition. It is possible that the working memory resources that support learning from gesture in a novel math task are quite distinct from the resources that support extracting information from gesture during discourse. However, it is also possible that our study was not sensitive to potential effects of kinesthetic working memory as the distribution of scores for kinesthetic WMC in our sample was non-normal and had a limited range.

Prior work has shown that WMC predicts mathematical ability (Alloway & Alloway, 2010; Gathercole & Pickering, 2000; Jarvis & Gathercole, 2003), and that gesture at instruction generally improves mathematical learning (Cook et al., 2013; Cook et al., 2016; Novack, Congdon, Hemani-Lopez, & Goldin-Meadow, 2014). This study is the first to demonstrate that gesture may not benefit all individuals equally. These results are important for enhancing theoretical understanding of how gestures are processed, but are also critical in informing instructional practice and interventions.

The findings reported here have implications for personalizing instruction. Because individuals with high visuospatial WMC are particularly likely to benefit when instruction includes gesture, and individuals with greater verbal WMC may benefit when instruction does not include gesture, the findings suggest that gestures may be particularly beneficial for learners with low verbal working memory but high visuospatial working memory. One set of learners that is likely to have this cognitive profile is learners with Developmental Language Disorder. Children with Developmental Language Disorder demonstrate worse mathematical performance than typically developing children, and this performance difference is accounted for by lower verbal working memory abilities (Fyfe, Matz, Hunt & Alibali, 2019). Interestingly, these same children perform similarly to typically developing children on a visual pattern task. Thus, learners with Developmental Language Disorder may be particularly likely to benefit from the additional visuospatial information that is afforded by gesture.

### Limitations and future directions

We did not find a significant difference in performance across our two instructional conditions, which is inconsistent with the prior literature. However, these data were not collected to optimally test for differences across instructional groups. Moreover, in all models combining the data from the two studies, performance in the gesture group was better than performance in the no-gesture group. It is possible that there is no overall benefit to gesture in this task, however, we believe this is unlikely given other ongoing work in our labs using the same task. It is also possible that the lack of an effect is due to the non-random sampling procedure or due to sampling variability expected when studying small effects without high-powered designs.

It is unclear exactly how visuospatial working memory supports learning with gesture. One possibility is that gesture and visuospatial working memory work together to enhance encoding of visuospatial information (Alibali, Crooks, & McNeil, 2018; Crooks & Alibali, 2013). The external support of gesture at instruction may work with visuospatial working memory to enhance the learner’s ability to mentally represent problem features. Previous work has shown that children’s encoding abilities are related to their strategy use (McNeil & Alibali, 2004). Future work might examine if there is a relationship between individual differences in WMC and encoding of information with or without gesture to assess the possibility that encoding mediates the relationship between WMC and learning.

Spatial ability is comprised of both spatial visualization and spatial reasoning, and has been found to uniquely predict STEM expertise above and beyond verbal and mathematical abilities (Lubinski, 2010; Wai et al., 2009). While we only used one task to tap into visuospatial working memory, future work may benefit from including multiple measures of spatial thinking in addition to measures of visuospatial WMC to attempt to gain a better understanding of the relationship between spatial ability, visuospatial working memory and mathematical learning with gesture.

## Conclusions

We found that visuospatial WMC predicted mathematical equivalence learning with gesture at instruction, even after controlling for composite ACT score. When gesture was not present at instruction, verbal working memory significantly predicted learning, after controlling for composite ACT score. These findings are the first to demonstrate a relationship between individual differences in WMC and learning with gesture in adults. This work has important implications for both educational and clinical settings and offers insight into specific populations that might benefit most from gesture as additional support during instruction.Sweller et al. 1998

## Supplementary information

**Additional file 1.** Supplementary Materials

## Data Availability

The datasets generated and analyzed during the current study are available on Open Science Framework, [https://osf.io/wh92e/]. Task materials as well as Supplementary Materials are also available.

## Abbreviations

A-MARS: Abbreviated Math Anxiety Rating Scale
VIF: Variance inflation factor
WMC: Working Memory Capacity
