# Supplementary material for: Gesture during math instruction specifically benefits learners with high visuospatial working memory capacity
Source: Cogn Res Princ Implic. 2020 Jun 9;5:27. doi: 10.1186/s41235-020-00215-8 (PMC7283399; doi:10.1186/s41235-020-00215-8)
Supplement: Supplementary file 1 — Additional file 1. Supplementary Materials [file 41235_2020_215_MOESM1_ESM.docx]

Supplementary Materials

This supplementary material contains model results from all analyses conducted on the data that are referenced in the paper.

Preregistered, full models of posttest and transfer performance included 3 way interactions between types of working memory, for Gesture and No Gesture Conditions, with separate analyses for Posttest and Transfer.

To see complete preregistrations from both studies in this paper, go to <https://osf.io/wh92e/>.

Supplemental Table 1

| *Preregistered Posttest Model - Gesture* |  |  |  |  |  |
| --- | --- | --- | --- | --- | --- |
| Fixed Effect | Estimate | Std. Error | *z* value | *p* value |  |
| (Intercept) | 1.91808 | 0.30211 | 6.349 | 2.17E-10 | *** |
| Composite ACT | 0.88408 | 0.23956 | 3.69 | 0.000224 | *** |
| VisWM | 0.43845 | 0.24589 | 1.783 | 0.074573 | . |
| VWM | -0.1266 | 0.23474 | -0.539 | 0.589664 |  |
| KWM | 0.05037 | 0.22441 | 0.224 | 0.822413 |  |
| VisWM:VWM | 0.03677 | 0.21681 | 0.17 | 0.865334 |  |
| VisWM:KWM | 0.03517 | 0.22616 | 0.156 | 0.876406 |  |
| VWM:KWM | -0.2011 | 0.2229 | -0.902 | 0.366952 |  |
| VisWM:VWM:KWM | 0.04252 | 0.2307 | 0.184 | 0.853772 |  |
| *Note*. **p*<.05; ***p*<.01; ****p*<.0001 |  |  |  |  |  |

registered.model.ge.post.glmer <- glmer( correct ~ Composite_S + VPT_Score_S*VWM_Span_S*KWM_Span_S + (1|SubjectID) + (1| Stimulus), data = subset(cg.data, Group == "GE" & Portion == "Post"), control = glmerControl(optimizer = "bobyqa", optCtrl=list(maxfun=20000)), family = "binomial")

Supplemental Table 2

| *Preregistered Transfer Test Model - Gesture* | |  |  |  |  |  |
| --- | --- | --- | --- | --- | --- | --- |
| Fixed Effect | Estimate | | Std. Error | *z* value | *p* value |  |
| (Intercept) | 0.86487 | | 0.33022 | 2.619 | 0.00882 | ** |
| Composite ACT | 0.48293 | | 0.1889 | 2.557 | 0.01057 | * |
| VisWM | 0.36441 | | 0.19661 | 1.853 | 0.06381 | . |
| VWM | 0.17083 | | 0.18448 | 0.926 | 0.35443 |  |
| KWM | 0.22686 | | 0.18116 | 1.252 | 0.21046 |  |
| VisWM:VWM | -0.06473 | | 0.17045 | -0.38 | 0.70411 |  |
| VisWM:KWM | -0.31811 | | 0.17084 | -1.862 | 0.06259 | . |
| VWM:KWM | 0.04801 | | 0.17682 | 0.272 | 0.78601 |  |
| VisWM:VWM:KWM | -0.22349 | | 0.18131 | -1.233 | 0.2177 |  |
| *Note*. **p*<.05; ***p*<.01 |  | |  |  |  |  |
|  |  | |  |  |  |  |

registered.model.ge.tran.glmer <- glmer( correct ~ Composite_S + VPT_Score_S*VWM_Span_S*KWM_Span_S + (1|SubjectID) + (1| Stimulus), data = subset(cg.data, Group == "GE" & Portion == "Tran"), control = glmerControl(optimizer = "bobyqa", optCtrl=list(maxfun=20000)), family = "binomial")

Supplemental Table 3

| *Preregistered Posttest Model - No Gesture* |  |  |  |  |  |
| --- | --- | --- | --- | --- | --- |
| Fixed Effect | Estimate | Std. Error | *z* value | *p* value |  |
| (Intercept) | 1.45436 | 0.32614 | 4.459 | 8.22E-06 | *** |
| Composite ACT | 0.47034 | 0.22394 | 2.1 | 0.0357 | * |
| VisWM | -0.01487 | 0.21083 | -0.071 | 0.9438 |  |
| VWM | 0.35317 | 0.23794 | 1.484 | 0.1377 |  |
| KWM | 0.26826 | 0.20139 | 1.332 | 0.1828 |  |
| VisWM:VWM | 0.27665 | 0.21863 | 1.265 | 0.2057 |  |
| VisWM:KWM | 0.079 | 0.20155 | 0.392 | 0.6951 |  |
| VWM:KWM | -0.02546 | 0.28284 | -0.09 | 0.9283 |  |
| VisWM:VWM:KWM | 0.05991 | 0.18475 | 0.324 | 0.7457 |  |
| *Note*. **p*<.05; ****p*<.0001 |  |  |  |  |  |

registered.model.ng.post.glmer <- glmer( correct ~ Composite_S + VPT_Score_S*VWM_Span_S*KWM_Span_S + (1|SubjectID) + (1| Stimulus), data = subset(cg.data, Group == "NG" & Portion == "Post"), control = glmerControl(optimizer = "bobyqa", optCtrl=list(maxfun=20000)), family = "binomial")

Supplemental Table 4

| *Preregistered Transfer Test Model - No Gesture* | | |  |  |  |  |
| --- | --- | --- | --- | --- | --- | --- |
| Fixed Effect | | Estimate | Std. Error | *z* value | *p* value |  |
| (Intercept) | 0.46681 | | 0.3984 | 1.172 | 0.2413 |  |
| Composite ACT | 0.70212 | | 0.17681 | 3.971 | 7.16E-05 | *** |
| VisWM | 0.05219 | | 0.16554 | 0.315 | 0.7526 |  |
| VWM | 0.28682 | | 0.18235 | 1.573 | 0.1157 |  |
| KWM | 0.04609 | | 0.15493 | 0.297 | 0.7661 |  |
| VisWM:VWM | 0.42055 | | 0.17805 | 2.362 | 0.0182 | * |
| VisWM:KWM | -0.10565 | | 0.15535 | -0.68 | 0.4964 |  |
| VWM:KWM | -0.13904 | | 0.21843 | -0.637 | 0.5244 |  |
| VisWM:VWM:KWM | 0.02694 | | 0.13888 | 0.194 | 0.8462 |  |
| *Note*. **p*<.05; ****p*<.0001 |  | |  |  |  |  |

registered.model.ng.tran.glmer <- glmer( correct ~ Composite_S + VPT_Score_S*VWM_Span_S*KWM_Span_S + (1|SubjectID) + (1| Stimulus), data = subset(cg.data, Group == "NG" & Portion == "Tran"), control = glmerControl(optimizer = "bobyqa", optCtrl=list(maxfun=20000)), family = "binomial")

Models exploring how math anxiety is related to performance looked at whether math anxiety predicted performance in either instructional group, and whether including math anxiety as a factor eliminated the observed patterns of performance. There was no evidence that math anxiety was related to performance in this task. Furthermore, including math anxiety in our models did not change the overall pattern of findings.

Supplemental Table 5

| *Including A_MARS in Full Posttest Model - Gesture* | | |  |  |  |
| --- | --- | --- | --- | --- | --- |
| Fixed Effects | Estimate | Std. Error | *z* value | *p* value |  |
| (Intercept) | 1.88002 | 0.29184 | 6.442 | 1.18E-10 | *** |
| Composite ACT | 0.85894 | 0.24911 | 3.448 | 0.000565 | *** |
| A_MARS | 0.02466 | 0.23355 | 0.106 | 0.9159 |  |
| VisWM | 0.44884 | 0.23659 | 1.897 | 0.057808 | . |
| VWM | -0.09273 | 0.18805 | -0.493 | 0.621946 |  |
| *Note*. ****p*<.0001 |  |  |  |  |  |

GE.post.main.mars.glmer <- glmer( correct ~ Composite_S + MARS_Total_S + VPT_Score_S + VWM_Span_S + (1|SubjectID) + (1| Stimulus), data=subset(cg.data, Group=="GE" & Portion=="Post"), control = glmerControl(optimizer = "bobyqa", optCtrl=list(maxfun=20000)), family = "binomial")

Supplemental Table 6

| *Including A_MARS in Full Transfer Test Model - Gesture* | | |  |  |  |
| --- | --- | --- | --- | --- | --- |
| Fixed Effects | Estimate | Std. Error | *z* value | *p* value |  |
| (Intercept) | 0.6935 | 0.3253 | 2.132 | 0.033 | * |
| Composite ACT | 0.4123 | 0.2029 | 2.032 | 0.0421 | * |
| A_MARS | -0.1438 | 0.1943 | -0.74 | 0.4591 |  |
| VisWM | 0.4145 | 0.1946 | 2.129 | 0.0332 | * |
| VWM | 0.0244 | 0.1534 | 0.159 | 0.8736 |  |
| *Note*. **p*<.05 |  |  |  |  |  |

GE.tran.main.mars.glmer <- glmer( correct ~ Composite_S + MARS_Total_S + VPT_Score_S + VWM_Span_S + (1|SubjectID) + (1| Stimulus), data=subset(cg.data, Group=="GE" & Portion=="Tran"), control = glmerControl(optimizer = "bobyqa", optCtrl=list(maxfun=20000)), family = "binomial")

Supplemental Table 7

| *Including A_MARS in Posttest Model with Visualspatial and Verbal Working Memory - No Gesture* | | |  |  |  |
| --- | --- | --- | --- | --- | --- |
| Fixed Effects | Estimate | Std. Error | *z* value | *p* value |  |
| (Intercept) | 1.624345 | 0.319878 | 5.078 | 3.81E-07 | *** |
| Composite ACT | 0.224213 | 0.220625 | 1.016 | 0.3095 |  |
| A_MARS | -0.276777 | 0.177068 | -1.563 | 0.118 |  |
| VisWM | 0.005621 | 0.199464 | 0.028 | 0.9775 |  |
| VWM | 0.447234 | 0.202009 | 2.214 | 0.0268 | * |
| *Note*. **p*<.05; ****p*<.0001 |  |  |  |  |  |

NG.post.main.mars.glmer <- glmer( correct ~ Composite_S + MARS_Total_S + VPT_Score_S + VWM_Span_S + (1|SubjectID) + (1| Stimulus), =subset(cg.data, Group=="NG" & Portion=="Post"), control = glmerControl(optimizer = "bobyqa", optCtrl=list(maxfun=20000)), family = "binomial")

Supplemental Table 8

| *Including A_MARS in Transfer Test Model with Visualspatial and Verbal Working Memory - No Gesture* | | |  |  |  |
| --- | --- | --- | --- | --- | --- |
| Fixed Effects | Estimate | Std. Error | *z* value | *p* value |  |
| (Intercept) | 0.553909 | 0.396081 | 1.398 | 0.162 |  |
| Composite ACT | 0.607358 | 0.179184 | 3.39 | 0.0007 | *** |
| A_MARS | -0.114873 | 0.141407 | -0.812 | 0.4166 |  |
| VisWM | 0.007204 | 0.161238 | 0.045 | 0.9644 |  |
| VWM | 0.361415 | 0.155829 | 2.319 | 0.0204 | * |
| *Note*. **p*<.05; ****p*<.0001 |  |  |  |  |  |

NG.tran.main.mars.glmer <- glmer( correct ~ Composite_S + MARS_Total_S + VPT_Score_S + VWM_Span_S + (1|SubjectID) + (1| Stimulus), data=subset(cg.data, Group=="NG" & Portion=="Tran"), control = glmerControl(optimizer = "bobyqa", optCtrl=list(maxfun=20000)), family = "binomial")

The gesture attitudes scales was broken down into 6 subscales measuring separable constructs based on communication from the original creators of the scale (Personal communication, Yeo, Feb 21, 2018). These scales were:

Positive Effect of Gesture in Instruction (PE)

Gesture Can Distract (DIS)

Gesture Matches Help Learning (MAL)

Gesture Mismatches Help Understanding (MAU)

Gesture is Unconscious (UNC)

Gesture Can be Controlled (CON)

We considered whether any of these variables was related to performance (combining posttest and transfer) in models that also included ACT. None of the constructs was related to performance, in either the gesture or no gesture condition.

Supplemental Table 9

| *Gesture Attitudes: PE (Benefit) - Gesture* | |  |  |  |  |
| --- | --- | --- | --- | --- | --- |
| Fixed Effects | Estimate | Std. Error | *z* value | *p* value |  |
| (Intercept) | 1.474643 | 0.237 | 6.222 | 4.90E-10 | *** |
| Composite ACT | 0.900908 | 0.15304 | 5.887 | 3.94E-09 | *** |
| PE | 0.009006 | 0.138311 | 0.065 | 0.948 |  |
| *Note*. ****p*<.0001 |  |  |  |  |  |

Supplemental Table 10

| *Gesture Attitudes: DIS (Distract) - Gesture* | |  |  |  |  |
| --- | --- | --- | --- | --- | --- |
| Fixed Effects | Estimate | Std. Error | *z* value | *p* value |  |
| (Intercept) | 1.4752589 | 0.2375043 | 6.212 | 5.25E-10 | *** |
| Composite ACT | 0.9007032 | 0.154286 | 5.838 | 5.29E-09 | *** |
| DIS | -0.0003797 | 0.1376005 | -0.003 | 0.998 |  |
| *Note*. ****p*<.0001 |  |  |  |  |  |

Supplemental Table 11

| *Gesture Attitudes: MAL (Redundant) - Gesture* | |  |  |  |  |
| --- | --- | --- | --- | --- | --- |
| Fixed Effects | Estimate | Std. Error | z value | p value |  |
| (Intercept) | 1.4637 | 0.23779 | 6.156 | 7.48E-10 | *** |
| Composite ACT | 0.88316 | 0.15703 | 5.624 | 1.87E-08 | *** |
| MAL | 0.06971 | 0.1469 | 0.475 | 0.635 |  |
| *Note*. ****p*<.0001 |  |  |  |  |  |

Supplemental Table 12

| *Gesture Attitudes: MAU (Complementarity) - Gesture* | |  |  |  |  |
| --- | --- | --- | --- | --- | --- |
| Fixed Effects | Estimate | Std. Error | *z* value | *p* value |  |
| (Intercept) | 1.4832 | 0.2355 | 6.298 | 3.01E-10 | *** |
| Composite ACT | 0.922 | 0.1519 | 6.069 | 1.28E-09 | *** |
| MAU | -0.1748 | 0.1287 | -1.358 | 0.174 |  |
| *Note*. ****p*<.0001 |  |  |  |  |  |

Supplemental Table 13

| *Gesture Attitudes: UNC (Unconscious) - Gesture* | |  |  |  |  |
| --- | --- | --- | --- | --- | --- |
| Fixed Effects | Estimate | Std. Error | *z* value | *p* value |  |
| (Intercept) | 1.4531 | 0.2375 | 6.119 | 9.41E-10 | *** |
| Composite ACT | 0.8726 | 0.1555 | 5.61 | 2.02E-08 | *** |
| UNC | -0.1108 | 0.1365 | -0.812 | 0.417 |  |
| *Note*. ****p*<.0001 |  |  |  |  |  |

Supplemental Table 14

| *Gesture Attitudes: CON (Controlled) - Gesture* | |  |  |  |  |
| --- | --- | --- | --- | --- | --- |
| Fixed Effects | Estimate | Std. Error | *z* value | *p* value |  |
| (Intercept) | 1.46754 | 0.23698 | 6.193 | 5.91E-10 | *** |
| Composite ACT | 0.88675 | 0.15493 | 5.723 | 1.04E-08 | *** |
| CON | 0.07796 | 0.14921 | 0.523 | 0.601 |  |
| *Note*. ****p*<.0001 |  |  |  |  |  |

cg.gest.PE.GE.glmer <- glmer( correct ~ Composite_S + PE_S + (1|SubjectID) + (1| Stimulus), data=subset(cg.data, Group=="GE"), control = glmerControl(optimizer = "bobyqa", optCtrl=list(maxfun=20000)), family = "binomial")

*Model script for tables 9-14 are identical aside from the specific gesture attitudes variable being examined.*

Supplemental Table 15

| *Gesture Attitudes: PE (Benefit) - No Gesture* | |  |  |  |  |
| --- | --- | --- | --- | --- | --- |
| Fixed Effects | Estimate | Std. Error | *z* value | *p* value |  |
| (Intercept) | 1.1639 | 0.2545 | 4.573 | 4.80E-06 | *** |
| Composite ACT | 0.6203 | 0.1372 | 4.52 | 6.18E-06 | *** |
| PE | -0.1712 | 0.1464 | -1.169 | 0.242 |  |
| *Note*. ****p*<.0001 |  |  |  |  |  |

Supplemental Table 16

| *Gesture Attitudes: DIS (Distract) - No Gesture* | |  |  |  |  |
| --- | --- | --- | --- | --- | --- |
| Fixed Effects | Estimate | Std. Error | *z* value | *p* value |  |
| (Intercept) | 1.1606 | 0.2553 | 4.546 | 5.47E-06 | *** |
| Composite ACT | 0.6256 | 0.1385 | 4.518 | 6.25E-06 | *** |
| DIS | 0.1305 | 0.152 | 0.859 | 0.39 |  |
| *Note*. ****p*<.0001 |  |  |  |  |  |

Supplemental Table 17

| *Gesture Attitudes: MAL (Redundant) - No Gesture* | |  |  |  |  |
| --- | --- | --- | --- | --- | --- |
| Fixed Effects | Estimate | Std. Error | *z* value | *p* value |  |
| (Intercept) | 1.1961 | 0.2554 | 4.684 | 2.82E-06 | *** |
| Composite ACT | 0.6231 | 0.1378 | 4.522 | 6.14E-06 | *** |
| MAL | 0.1426 | 0.1464 | 0.974 | 0.33 |  |
| *Note*. ****p*<.0001 |  |  |  |  |  |

Supplemental Table 18

| *Gesture Attitudes: MAU (Complementarity) - No Gesture* | | |  |  |  |
| --- | --- | --- | --- | --- | --- |
| Fixed Effects | Estimate | Std. Error | *z* value | *p* value |  |
| (Intercept) | 1.17584 | 0.25503 | 4.611 | 4.02E-06 | *** |
| Composite ACT | 0.6095 | 0.13894 | 4.387 | 1.15E-05 | *** |
| MAU | -0.05554 | 0.16436 | -0.338 | 0.735 |  |
| *Note*. ****p*<.0001 |  |  |  |  |  |

Supplemental Table 19

| *Gesture Attitudes: UNC (Unconscious) - No Gesture* | |  |  |  |  |
| --- | --- | --- | --- | --- | --- |
| Fixed Effects | Estimate | Std. Error | *z* value | *p* value |  |
| (Intercept) | 1.135 | 0.2546 | 4.459 | 8.25E-06 | *** |
| Composite ACT | 0.6106 | 0.1353 | 4.512 | 6.43E-06 | *** |
| UNC | 0.2509 | 0.1591 | 1.577 | 0.115 |  |
| *Note*. ****p*<.0001 |  |  |  |  |  |

Supplemental Table 20

| *Gesture Attitudes: CON (Controlled) - No Gesture* | |  |  |  |  |
| --- | --- | --- | --- | --- | --- |
| Fixed Effects | Estimate | Std. Error | *z* value | *p* value |  |
| (Intercept) | 1.18848 | 0.25534 | 4.654 | 3.25E-06 | *** |
| Composite ACT | 0.58445 | 0.14425 | 4.052 | 5.08E-05 | *** |
| CON | 0.09833 | 0.14363 | 0.685 | 0.494 |  |
| *Note*. ****p*<.0001 |  |  |  |  |  |

cg.gest.PE.NG.glmer <- glmer( correct ~ Composite_S + PE_S + (1|SubjectID) + (1| Stimulus),

data=subset(cg.data, Group=="NG"), control = glmerControl(optimizer = "bobyqa", optCtrl=list(maxfun=20000)), family = "binomial")

*Model syntax for tables 15-20 are identical aside from the specific gesture attitudes variable being examined.*

Supplemental Table 21

| *Mean Performance On Measures By Group* | | |  |  | |  | |  | |  | |  |
| --- | --- | --- | --- | --- | --- | --- | --- | --- | --- | --- | --- | --- |
| Group | Performance (both post and transfer) | Composite ACT Score | | | VSWM | | VWM | | KWM | | Math Anxiety | |
| Gesture | 0.7047276 | 26.125 | | | 14.01562 | | 4.164062 | | 2.453125 | | 51.46875 | |
| No Gesture | 0.6915064 | 26.96875 | | | 13.25 | | 4.273438 | | 2.59375 | | 56.6875 | |

Supplemental Table 22

| *Combined Model of Post Test When Removing Math (and related) Majors* | | | | |  |
| --- | --- | --- | --- | --- | --- |
|  | Estimate | Std. Error | *z* value | *p* value |  |
| (Intercept) | 2.087 | 0.3141 | 6.645 | 3.04E-11 | *** |
| Composite ACT | 0.5192 | 0.1793 | 2.896 | 0.00378 | ** |
| GroupNG | -0.5155 | 0.2918 | -1.766 | 0.07731 | . |
| VisWM | 0.6287 | 0.2433 | 2.584 | 0.00976 | ** |
| VWM | -0.1333 | 0.2107 | -0.633 | 0.52681 |  |
| GroupNG:VisWM | -0.6835 | 0.2995 | -2.282 | 0.02248 | * |
| GroupNG:VWM | 0.5523 | 0.3107 | 1.778 | 0.07547 | . |
| *Note.* *p<.05; **p<.001; ***p<.0001 | |  |  |  |  |

Supplemental Table 23

| *Combined Model of Transfer Test When Removing Math (and related) Majors* | | | | |  |
| --- | --- | --- | --- | --- | --- |
|  | Estimate | Std. Error | *z* value | *p* value |  |
| (Intercept) | 0.9095 | 0.39227 | 2.319 | 0.0204 | * |
| Composite ACT | 0.61967 | 0.14534 | 4.264 | 2.01E-05 | *** |
| GroupNG | -0.36435 | 0.23202 | -1.57 | 0.1163 |  |
| VisWM | 0.32181 | 0.19079 | 1.687 | 0.0917 | . |
| VWM | -0.07515 | 0.16604 | -0.453 | 0.6508 |  |
| GroupNG:VisWM | -0.38659 | 0.23681 | -1.632 | 0.1026 |  |
| GroupNG:VWM | 0.50141 | 0.24266 | 2.066 | 0.0388 | * |
| *Note.* *p<.05; ***p<.0001 |  |  |  |  |  |
